# *Pneumocystis jirovecii* Colonization in Preterm Newborns With Respiratory Distress Syndrome

**DOI:** 10.1093/infdis/jiab209

**Published:** 2021-04-15

**Authors:** Magdalena Szydłowicz, Barbara Królak-Olejnik, Sergio L Vargas, Żaneta Zajączkowska, Dorota Paluszyńska, Anna Szczygieł, Olga Matos, Andrzej B Hendrich, Marta Kicia

**Affiliations:** 1 Department of Biology and Medical Parasitology, Wroclaw Medical University, Wroclaw, Poland; 2 Department and Clinic of Neonatology, Wroclaw Medical University, Wroclaw, Poland; 3 Instituto de Ciencias Biomédicas, Facultad de Medicina Universidad de Chile, Santiago, Chile; 4 Unit of Medical Parasitology, Global Health and Tropical Medicine, Instituto de Higiene e Medicina Tropical, Universidade Nova de Lisboa, Lisbon, Portugal

**Keywords:** *Pneumocystis jirovecii*, preterm infants, vertical transmission, respiratory distress syndrome, bronchopulmonary dysplasia

## Abstract

We describe the prevalence of *Pneumocystis jirovecii* in mother-infant pairs of very low birth weight newborns <32 weeks gestation. Molecular and microscopic methods were used for detection of *P. jirovecii* in patients’ specimens. *Pneumocystis* DNA was detected in 8 nasopharyngeal aspirates (14%) of 56 newborns and in 7 oral washes (21%) of 34 mothers. *Pneumocystis* detection immediately after birth suggests the possibility of its transplacental transmission. Compared to noncolonized infants, more frequent occurrence of bronchopulmonary dysplasia was seen in colonized infants (*P* = .02), suggesting a potential clinical importance of this pathogen in abnormal lung development.

Previous reports suggest that *Pneumocystis jirovecii* may be transmitted from mother to infant [1]. Moreover, a significant increase of respiratory distress syndrome has been documented in preterm infants colonized with *P. jirovecii* [2], emphasizing the need for research to identify whether this fungus has a pathogenic role in newborns. Relatively high prevalence of *P. jirovecii* among pregnant women in the third trimester [3] may pose a risk of transplacental transmission. Colonized mothers may also act as a postpartum aerial reservoir of this pathogen for their immunologically naive newborns [1–3]. The aim of this study was to investigate the prevalence of *P. jirovecii* among newborns with respiratory distress syndrome, born prematurely, and/or with very low birth weight. Respiratory specimens were also collected from mothers to investigate genetic homology with isolates from their newborns.

## METHODS

Respiratory specimens from human immunodeficiency virus (HIV)-negative (1) preterm infants (n = 56) who had been under the care of the Department and Clinic of Neonatology, Wroclaw Medical University, Wroclaw, Poland, and (2) their mothers (n = 34; specimens from 8 mothers were not available) were examined between January 2018 and June 2019. Nasopharyngeal aspirates were obtained from newborns immediately after birth (approximately 10–15 minutes after birth), and oral washes were collected from mothers shortly after labor, as described previously [2, 4]. In the case of 6 infants, a resampling from the respiratory tract for other diagnostic tests was taken within 3.5–22 weeks after delivery, which was used to check the presence of *Pneumocystis* again. Inclusion criteria for newborns were (1) birth weight <1500 g and/or gestational age <32 weeks, and (2) respiratory distress syndrome, defined as respiratory failure in the first hours of life of a preterm newborn presumably caused by lung surfactant deficiency. Diagnosis of respiratory distress syndrome was made based on clinical symptoms, chest X-ray, and blood gas analysis results. All patients during stabilization at the delivery room were started on nasal continuous positive airway pressure (nCPAP) as the initial respiratory support. In cases with (1) arterial blood pH < 7.20, (2) Paco_2_ ≥ 60 mmHg, (3) Sao_2_ < 90% at O_2_ concentration above 30% and nCPAP of 6–10 cmH_2_O, and (4) persistent or severe apnea, respiratory failure was diagnosed. According to European guidelines [5], a less invasive surfactant administration method was used in babies who were worsening when Fio_2_ > 0.30 on nCPAP pressure of at least 6 cmH_2_O. After stabilization, mechanical ventilation was used in babies with respiratory distress syndrome when noninvasive methods of respiratory support had failed. When the intubation was required as part of stabilization, surfactant was given immediately. Demographic, clinical, and laboratory data were recorded upon inclusion for all study participants. Informed consent, approved by the Human Research Ethics Committee of Wroclaw Medical University (agreement No. KB-725/2017), was obtained from parents or statutory representatives of infants, as well as from mothers whose samples were included in the study.

Samples collected from all patients were tested by both molecular and microscopic techniques. Molecular detection was performed by nested polymerase chain reaction (PCR) amplifying partial sequence of *P. jirovecii mtLSU* rRNA gene, followed by sequencing and phylogenetic analyses [6, 7]. Nested PCR was repeated 3 times for each sample, and those with confirmed *Pneumocystis* DNA presence in at least 1 independent analysis were considered positive. Microscopic examination of fixed smear slides of centrifuged respiratory specimens was performed using a direct immunofluorescence assay (MONOFLUO *Pneumocystis jirovecii* IFA Test Kit; BioRad).

Fisher exact test and Student *t* test were used to compare variables between *Pneumocystis-*positive and -negative individuals. The duration of the oxygen therapy (with time to therapy termination as outcome) was analyzed using the Kaplan-Meier method, censoring those infants who died and reweighting by inverse probability of censoring weights. This enabled consideration of the Kaplan-Meier curves as representing the duration of oxygen therapy in a group of infants who all survived. The duration of oxygen therapy was compared between colonized and noncolonized patients with the use of the log rank test for univariate analysis in the R environment (R version 4.0.4). A value of *P* < .05 was considered significant. The analysis of the duration of oxygen therapy was performed with the use of the R packages *pec, prodlim*, and *RISCA* [8–10]. Due to the exploratory nature of the study, no adjustments have been made for multiple comparisons.

## RESULTS

Basic clinical and demographic characteristics of studied infants are shown in Table 1. *P. jirovecii* DNA was detected in 8 (14%) of 56 newborns immediately after birth and in 7 (21%) of 34 mothers, but no matched mother-newborn pairs were detected. Children of 8 mothers from whom specimens were not available were *Pneumocystis* negative. Among infants tested more than once, 2 were positive in 2 samplings (Table 2). Immunofluorescence microscopy confirmed cysts in 75% (6/8) infants immediately after birth (Table 2) and 57% (4/7) mothers (data not shown) with positive PCR result and all of them were considered colonized (less than 5 cysts per slide). No cysts were observed in PCR-negative samples.

**Table 1.** Comparison of Clinical and Demographic Characteristics of Infants With (Positive) and Without (Negative) *Pneumocystis jirovecii* Infection Immediately After Birth

Characteristic	*Pneumocystis jirovecii*		*P* Value
	Positive (n = 8)	Negative (n = 48)	
Sex, No. of patients (%)			
Male	5 (62.5)	30 (62.5)	1
Female	3 (37.5)	18 (37.5)	1
Birth weight, g	1320 (720–1940)	1270 (500–2200)	.34
Gestational age, wk	29 (24–34)	30 (25–33)	.73
MV, No. of patients (%)	3 (37.5)	21 (43.8)	1
MV duration, d	9 (9–30)	6 (2–46)	.45
nCPAP, No. of patients (%)	8 (100)	45 (94)	1
nCPAP duration, d	4.5 (1–16)	6 (1–43)	.39
Supplemental oxygen at 28 d, No. of patients (%)^a^	6 (75)	15 (35.7)	.06
Bronchopulmonary dysplasia, No. of patients (%)	6 (75)	14 (29)	.02^c^
Surfactant replacement therapy, No. of patients (%)	4 (50)	20 (42)	.71
Duration of hospitalization, d	44 (19–80)	45 (2–85)	.68
Type of delivery (%)^b^			
Cesarean delivery	6 (75)	44 (92)	.24
Vaginal delivery	2 (25)	4 (8)	.24
Maternal age, y	32 (22–37)	32 (18–43)	.54

Data represent median (range) unless otherwise indicated.

Abbreviations: MV, mechanical ventilation; nCPAP, nasal continuous positive airway pressure.

^a^Six *Pneumocystis-*negative infants who died below the age of 28 days were excluded.

^b^In a triplet pregnancy, the first infant was born by vaginal delivery and delay in subsequent births led to cesarean delivery.

^c^ *P* value < .05.

**Table 2.** *Pneumocystis* Diagnosis in Samples Collected From All Infants With Confirmed Colonization Throughout the Study Period

Patient No.	Sampling No.	*Pneumocystis* Diagnosis	
		Nested PCR	Microscopy
1	I	P	P
2	I	P	P
3	I	P	P
	II	P	P
4	I	P	P
	II	N	N
5^a,b^	I	N	N
	II	N	N
	III	P	N
6^a^	I	P	N
	II	P	P
	III	N	N
7	I	P	N
8	I	P	P
9	I	P	P

Abbreviations: N, negative; P, positive; PCR, polymerase chain reaction.

^a^Twin siblings.

^b^Infant not considered as a positive case immediately after birth because *Pneumocystis* was detected in the third sampling (20.7 weeks). The remaining 2 infants with more than 1 sample collected, tested *Pneumocystis-*negative at both samplings.

A significant association was demonstrated between colonization and bronchopulmonary dysplasia (*P* = .02). Median duration of oxygen therapy in colonized infants was 41.0 days (interquartile range [IQR], 31.0–55.0) and in noncolonized 19.0 days (IQR, 7.0–36.0; *P* = .07; Supplementary Figure 1). Anti-*Pneumocystis* treatment with sulfamethoxazole plus trimethoprim (5:1, co-trimoxazole; 2 doses of 100 mg/kg/day of sulfamethoxazole intravenously) was implemented in 1 patient due to persistent respiratory failure and prolonged requirement for mechanical ventilation. Seven infants died due to complications connected with extreme prematurity, but they all tested *Pneumocystis* negative.

Fourteen (33%) of the 42 pregnancies were multiple. Among them, *Pneumocystis* was recorded in 2 pairs of twins: in only 1 of the siblings in the first pair and in both infants in the second pair, that is one positive immediately after birth and the other one after discharge from the hospital (at the third sample collection after 20.7 weeks).

In most mothers’ samples *mtLSU* rRNA genotype 1 was detected, except in 1 case in which genotype 2 was present; in infants, genotype 1 was predominant (detected in 7 children), while genotypes 2 and 3 were recorded in 1 infant each. The isolates in the twin pair were genetically identical.

## Discussion

This study shows a significant relationship between *P. jirovecii* colonization and bronchopulmonary dysplasia—a common complication in very low gestational age newborn infants requiring supplemental oxygen for at least 28 days (Table 1). This is in accord with Rojas et al [2], although the difference in this study was not statistically significant. We also observed extended duration of oxygen therapy in *Pneumocystis-*colonized infants, and although this difference was not statistically significant, both of these results suggest that this fungus may contribute to the higher severity of respiratory distress syndrome in newborns and increase the duration of respiratory support. Possible explanations could be disruption of surfactant composition [11] and the increased mucus production associated with *Pneumocystis* colonization reported in infant lungs [12]. These data suggest a potential role of *Pneumocystis* colonization in bronchopulmonary dysplasia and prompt further research with a larger study group.

The prevalence of *Pneumocystis* among tested mothers (21%) was higher than in previous reports from women in the third trimester (5.4%–16.5%) [3, 13, 14]. Interestingly, in our study the prevalence of *Pneumocystis* was also higher among the group of mothers when compared to the infants (14%). The opposite was observed by Vera et al in full term infants [13], while none of the examined children was infected in the study by Garcia et al [14]. The slightly higher prevalence of *Pneumocystis* in mothers of very low birth weight preterm infants in our study compared to the series of term mother-infant pairs in the literature may suggest that colonization is associated with premature delivery. In this respect, the significance of *P. jirovecii* carriage in pregnant women requires further thorough analysis.

The scant evidence for transplacental transmission of *P. jirovecii* in humans provided by examinations of formalin-fixed tissues of aborted fetuses suggests the possible dissemination of the fungus from mother to fetus via the blood route [1]. The specimens in our study were collected several minutes after birth, and because the mother-infant skin-to-skin bonding contact procedure was not applied in our study, the transplacental route, rather than aerial, could have been a source of infection for these infants. The probability of such vertical transmission is additionally supported by the fact that cysts were observed in the first sampling in 6 of 8 PCR-positive infants’ specimens, thus the infection must have occurred sometime earlier. However, no matched mother-child pairs of infected individuals were recorded. It is possible that mothers were infected earlier during gestation, *Pneumocystis* was transmitted to the fetus and persisted until delivery, while the mother’s infection resolved spontaneously in the following weeks and therefore showed a negative result post partum. It is also possible that *Pneumocystis*-positive mothers were infected shortly before the specimen collection and the time frame was too short for their infants to become infected before delivery. The absence of matched pairs in our study could also be attributed to differences in types of collected specimens. It has been suggested that the number of organisms reaching the oral cavity may be insufficient for *Pneumocystis* detection in patients with low pulmonary fungal burden [15]. It can therefore be assumed that mothers of PCR-positive infants were also colonized, but we were unable to confirm this due to less sensitive diagnostic material.

Although *mtLSU* rRNA genotype 1 was the predominant in all samples investigated in this study, finding of genetically identical isolates in the twin pair cannot exclude sibling-to-sibling *Pneumocystis* transmission or a common infective source for both of them. On the other hand, *mtLSU* rRNA genotyping does not provide a sufficiently high discriminatory power for pathogen transmission analysis, thus it can also be assumed that there was another, external infective source for the second twin. Therefore, it should be borne in mind that children born prematurely are potentially at risk of various infections, including *Pneumocystis*, during infancy. Genotype 1 was also most commonly observed in the mothers’ specimens in the study of Vera et al [13], suggesting that this genotype may be prevalent during gestation. Pregnant women regularly attend outpatient clinics, which may promote the spread of the particular genotype in this group and can be further transmitted to their infants.

In conclusion, our study documents *Pneumocystis* DNA shortly after birth in very low birth weight premature infants that did not enter into close contact with their mothers, providing further support that *Pneumocystis* is highly common in premature infants and suggesting that blood-borne transmission via the transplacental route may be an alternative route for transmission of this fungus. *Pneumocystis* colonization in preterm infants may be associated with bronchopulmonary dysplasia, suggesting a potential clinical importance of this pathogen in abnormal lung development. Because prevalence of *Pneumocystis* among pregnant women and newborns is relatively high, this pathogen should be considered in the etiologic workup of newborns with respiratory disorders, especially in infants born prematurely.

## Supplementary Data

Supplementary materials are available at *The Journal of Infectious Diseases* online. Consisting of data provided by the authors to benefit the reader, the posted materials are not copyedited and are the sole responsibility of the authors, so questions or comments should be addressed to the corresponding author.

jiab209_suppl_Supplementary_Figure_S1

## Notes

***Acknowledgments.*** We thank Robin and Barbara Royle for their help in the language correction of the text and Krzysztof Kujawa from Statistical Analysis Centre of Wroclaw Medical University for assistance in statistical analysis of the results.

***Financial support.*** This work was financially supported by the Polish Ministry of Health subvention according to numbers STM.A060.20.093, STM.A060.20.105, and SUB.A300.19.015 from the IT Simple system of Wroclaw Medical University; and the University of Chile (grant number ENL30/19).

***Potential conflicts of interest.*** All authors: No reported conflicts of interest. All authors have submitted the ICMJE Form for Disclosure of Potential Conflicts of Interest. Conflicts that the editors consider relevant to the content of the manuscript have been disclosed.
