# Supplementary figures and images for: Pneumocystis jirovecii Colonization in Preterm Newborns With Respiratory Distress Syndrome
Source: J Infect Dis. 2021 Apr 15;225(10):1807–10. doi: 10.1093/infdis/jiab209 (PMC9113508; doi:10.1093/infdis/jiab209)

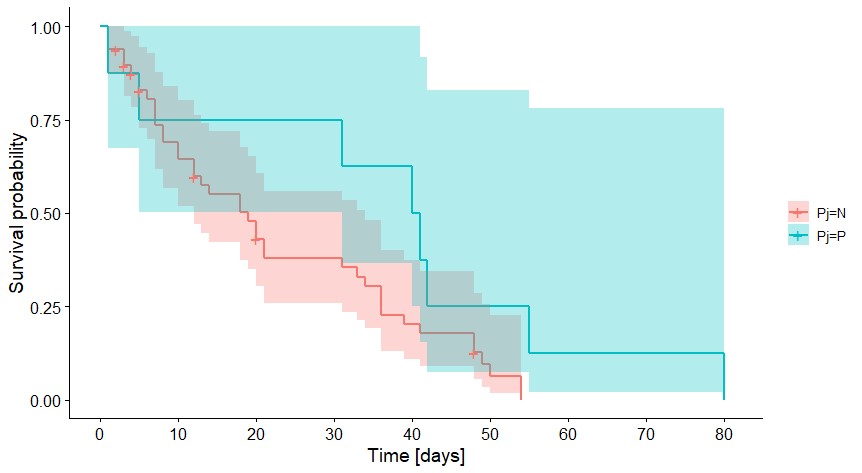

Supplement: jiab209_suppl_Supplementary_Figure_S1 [file jiab209_suppl_supplementary_figure_s1.jpeg]
